# Prevalence and Determinants of Malnutrition Among Adolescents in Delhi: A Rural-Urban Comparison Study

**DOI:** 10.7759/cureus.39135

**Published:** 2023-05-17

**Authors:** Aanchal Anand, Pragya Sharma

**Affiliations:** 1 Community Medicine, Andaman & Nicobar Islands Institute of Medical Sciences (ANIIMS), Port Blair, IND; 2 Community Medicine, Maulana Azad Medical College, New Delhi, IND

**Keywords:** urban and rural community, socio demographic factors, body mass index, malnutrition, adolescence

## Abstract

Background

Adolescence represents the period of transition from puberty to adulthood, encompassing development in the physical, cognitive, and psychosocial domains. Thus, this is a period of rapid growth, which is only second to that of infancy. Since the dietary patterns in this age group are influenced by many factors, adolescents have a higher tendency to remain malnourished.

Aim and objective

To find out the prevalence of and the socio-demographic factors associated with malnutrition among adolescents in a rural and an urban community of Delhi.

Materials and methods

This community-based cross-sectional study was conducted in rural and urban field practice areas under the aegis of the Department of Community Medicine, Maulana Azad Medical College, for the duration of one year. All eligible adolescents (10-19 years) residing in both study areas were included as the sampling frame. A total of 420 participants were enrolled in the study using the simple random sampling technique. All interviews were conducted face-to-face by the investigator to collect data on the nutritional status and socio-demographic variables of the study participants. The data were analyzed using SPSS version 26.0 (IBM Corp., Armonk, NY).

Results

The mean age of the participants in our study was found to be 15.65 ± 2.10 years. About 63% of males and 37% of females participated in the study. Participants from urban areas had a better socio-economic status, as 67.1% of participants were either in Class II or Class III of the modified BG Prasad Scale, vis-à-vis 36.6% of participants from rural areas. The overall prevalence of malnutrition was found to be 46% with overnutrition being more rampant than undernutrition.

Conclusion

The overall prevalence of malnutrition was 46% in the present study, out of which 18% were undernourished while 28% were over-nourished. The prevalence of undernutrition was approximately three times more in rural areas as compared to urban areas while the prevalence of obesity/overweight was more rampant in urban areas in comparison to rural areas.

## Introduction

Adolescence represents the period of transition from puberty to adulthood, encompassing development in the physical, cognitive, and psychosocial domains [1]. The World Health Organization (WHO) defines adolescents as any person between 10 and 19 years of age [2]. India is home to 253 million adolescents comprising 120 million girls and 133 million boys [3]. Our country’s sustained economic growth is dependent on its adolescents who are its demographic advantage and hence, it is important that they are well-nourished and healthy [4].

The critical period of adolescence also corresponds to diverse age-related changes that are influenced by social and cultural factors during this transition. Physically, the adolescent years are pivotal for both girls and boys as they achieve their maximum growth spurt during these years [5].

Good nutrition is a basic need, a human right, and fundamental to health and well-being at all ages and more so during adolescence [6]. Undernutrition and overnutrition represent extreme outliers on the bell curve of adiposity. While high tendencies of impaired cognitive development, short stature, poor educational achievement, and lower immunity leading to higher morbidity and mortality are the adverse effects of under-nutrition; adolescent obesity, on the other hand, is associated with serious health problems in adolescence and later adulthood, including a heightened risk of psychosocial morbidity, cardiovascular complications, type 1 and type 2 diabetes, premature death, and impaired social integration and stigmatization [7].

## Materials and methods

Study design

This was a* *community-based cross-sectional study.

Study area

The study was conducted in rural and urban field practice areas under the aegis of the Department of Community Medicine, Maulana Azad Medical College, New Delhi. The rural population was selected from Barwala village located in Narela Tehsil of North-West Delhi, with a population of 5100 comprising about 1020 adolescents as per the most recent survey conducted by field health workers. The urban population was chosen from the Delhi Gate area, Daryaganj, which comes under the Central Delhi District of New Delhi. The population of this area was 7700, comprising 1368 adolescents as per the most recent annual population demographic survey conducted by field health workers of an urban health center.

Study period

The study was conducted for the duration of one year with data collection between January 2020 and March 2020 (pre-lockdown) and August 2020 and April 2020 (post-lockdown). Due to the coronavirus (COVID) pandemic and lockdown, data collection was resumed only after restrictions were lifted and cases reduced, following COVID-appropriate behavior.

Study population

The study population consisted of eligible adolescents (10-19 years) residing in the study areas.

Inclusion Criteria

Those who were residents of the study area for at least one year.

Exclusion Criteria

Adolescents with chronic illnesses like tuberculosis and cancers and on special diets and restricted physical activity.

Sampling method

The simple random sampling method was adopted and random numbers generated by the random number table method were used to select study participants.

Ethical considerations

Approval was obtained from the Institutional Ethics Review Committee. The objective and procedure of the study were explained to the participants in the local language. Written informed consent/assent was taken from the participants as applicable. The provision of opting out from the study was kept open without any clause. The serial number instead of the name of the participant was used to ensure confidentiality regarding the identity of the participant.

Methodology

The areas were selected, as these are the field practice areas attached to the Department of Community Medicine, Maulana Azad Medical College as an Urban Health Center (UHC) and a Rural Health Training Centre (RHTC). Data were collected using a pre-tested semi-structured interview schedule. Pretesting was done in the Gokalpuri area of the North-East District attached to Maulana Azad Medical College.

The list of adolescents was taken from ASHA workers in that respective area and the total number of eligible participants based on the inclusion criteria were enlisted. Study participants were selected by simple random sampling technique using the random number table method and enrolled in the study. If the study subject was not present on the day of the visit, they were covered in the next visit. If a particular house was found to be locked on three consecutive visits, the study subject was dropped from the study and the next eligible participant was chosen. All interviews were conducted at adolescents' homes, ensuring the privacy of the subject in a separately requested room in their house for the duration of the interview by face-to-face interaction with the investigator to collect data on nutritional status and associated socio-demographic variables of the study participants.

Sample size calculation

The sample size was calculated using the formula:

 N= Z^2^_(1-α/2)_ *P(1-P)/d^2^ 

Where, N = sample size

 Z( 1- α/2) = 1.96 value of the standard normal variate corresponding to the level of significance α of 0.5

 d = specified absolute error on either side of the mean

 P = prevalence of malnutrition in Indian adolescents

Based on the previous study by Gupta A et al. [8], the prevalence of malnutrition among Indian adolescents was found to be P = 41.3% taking absolute error (d) = 5% and the sample size calculated was N = 378. Taking into account 10% of the non-response rate, an estimated total of 420 subjects (out of which 210 study subjects belonged to rural and 210 study subjects belonged to urban areas) were included in the study.

Study tool

A semi-structured interview schedule was used for data collection after pre-testing and pre-validation by the investigator, following which a physical examination of participants was conducted. The interview schedule was designed to elicit information on the identification and socio-demographic details of the participants and families and their education level and socio-economic status. After the interview, anthropometric measures, such as height and weight, were taken. BMI was calculated from the collected data. A general physical examination was also performed to detect conditions like pallor, icterus, lymphadenopathy, clubbing, edema, skin infections, etc. Pulse, respiratory rate, and temperature were recorded, and blood pressure was measured with a digital sphygmomanometer with accuracy to the nearest 1 mm of Hg.

Consent

The Institutional Ethics Committee, Maulana Azad Medical College, New Delhi, India, issued approval IEC/MAMC/70/05/2019/425. Written informed consent was obtained from the study participants after explaining to them the nature and purpose of the study.

Statistical analysis

Data were entered in Microsoft Excel (Microsoft Corporation, Redmond, WA), and analysis was done using Statistical Package for the Social Sciences (SPSS) PC version 26 (IBM Corp., Armonk, NY). Categorical variables were expressed in frequency and percentages. Quantitative variables are expressed in mean and standard deviation. The chi-square test was used to test differences in proportions. The mean difference between the groups was tested by the independent student's t-test and analysis of variance (ANOVA). A two-tailed p-value of <0.05 was considered statistically significant.

## Results

The study was conducted among a total of 420 subjects comprising 210 subjects each from both urban and rural areas. The mean age of the participants in our study was 15.65 ± 2.10 years.

Socio-demographic characteristics

Out of 210 subjects from rural areas, 11% were early adolescents (10-13 years), 36.1% were in mid-adolescence (14-16 years), and 52.9% were late adolescents (17-19 years) as per the WHO classification. However, in the urban areas, 22.4% of participants were early adolescents, 48.6% participants were mid adolescents and 29% were in late adolescence. Out of 420 subjects, 62.9% of males and 37.1% of females participated in the study. Comparing rural and urban areas, in rural areas, 69% of the participants were males and 31% were females in comparison to 56.7% males and 43.3% females of study participants in urban areas. The difference can be attributed to less participation by female subjects in rural areas.

More than half (53.8%) of the participants from rural areas were in middle school and an almost equal proportion (51%) of study subjects from urban areas were pursuing an intermediate level of education. The majority of study subjects from rural areas (91.9%) and urban areas (56.2%) were Hindus while the rest were Muslims. Among the participants from rural areas, only 9.5% were married, whereas all the study participants from urban areas were unmarried. Thus, as of date, almost one in 10 adolescents were married in rural areas, whereas in urban areas, no adolescent was found to be married, showing a strong sociocultural effect on the lives of adolescents.

The majority (60%) of caregivers of adolescents in rural areas were educated up to primary school; however, none was found to be educated beyond middle school as against more than half (55.7%) of the participant’s caregivers in urban areas being graduates and the rest were educated at least up to high school. The joint family system was more commonly (63%) seen in rural areas followed by a three-generation family (23%) and a few (14%) in nuclear families. On the contrary, an almost equal proportion of adolescents were staying in joint families (40%) or nuclear-type families (39%), whereas only one-fifth of the adolescents lived in a three-generation family (Table 1).

**Table 1:** Distribution of study participants on the basis of socio-demographic characteristics

Variables	Categories	Rural (n=210) ‘n’ (%)	Urban (n=210) ‘n’ (%)	Total (N=420) ‘n’ (%)
Age Category	10-13	23 (11.0)	47(22.4)	70 (16.6)
	14-16	76(36.1)	102(48.6)	178 (42.4)
	17-19	111(52.9)	61(29.0)	172 (41.0)
Gender	Male	145 (69.0)	119 (56.7)	264 (62.9)
	Female	65 (31.0)	91 (43.3)	156 (37.2)
Education Level	Illiterate	3(1.4)	0(0.0)	3 (0.7)
	Primary School	29(13.8)	9(4.3)	38 (9.0)
	Middle School	113(53.8)	19(9.0)	132 (31.4)
	High School	46(22.0)	75(35.7)	121 (28.9)
	Intermediate	19(9.0)	107(51.0)	126 (30.0)
Religion	Hindu	193 (91.9)	118 (56.2)	311 (74.0)
	Muslim	17 (8.1)	92 (43.8)	109 (26.0)
Marital status	Unmarried	190 (90.5)	210 (100.0)	400 (95.2)
	Married	20 (9.5)	0 (0.0)	20 (4.8)
Education level of their caregiver	Illiterate	36 (17.1)	0 (0.0)	36 (8.6)
	Primary School	126 (60.0)	0 (0.0)	126 (30.0)
	Middle School	48 (22.9)	0 (0.0)	48 (11.4)
	High School	0 (0.0)	7 (3.3)	7 (1.7)
	Intermediate	0 (0.0)	38 (18.1)	38 (9.0)
	Graduation	0 (0.0)	117 (55.7)	117 (27.9)
	Post-graduate & above	0 (0.0)	48 (22.9)	48 (11.4)
Type of family	Nuclear	29 (14.0)	82 (39.0)	111 (26.5)
	Joint	132 (63)	84 (40.0)	216 (51.5)
	Three Generation	48 (23)	44 (21.0)	92 (22.0)

**Figure 1:**
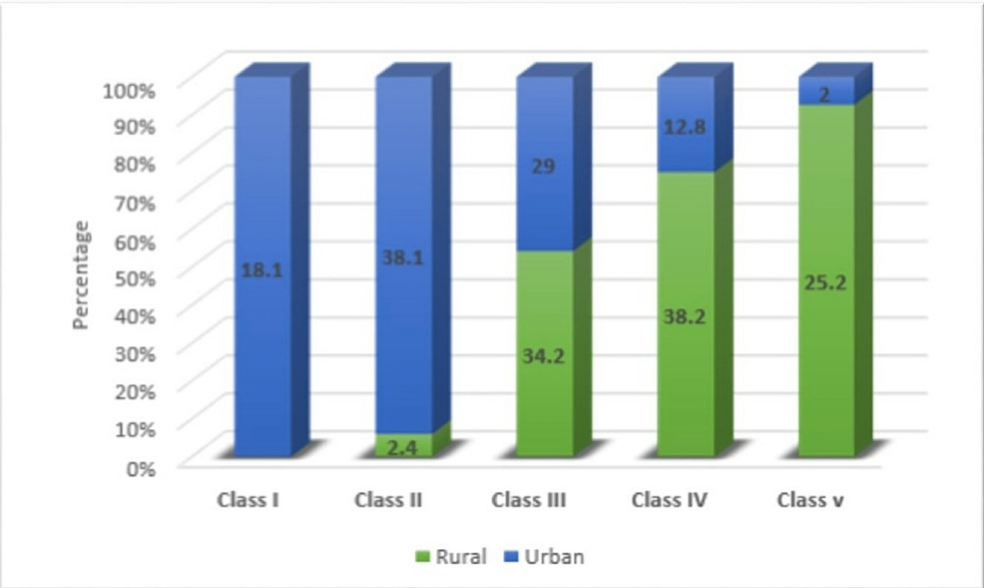
Distribution of study participants on the basis of the socioeconomic status of the family

The modified BG Prasad Scale was used to classify the participants based on their socioeconomic status. Almost two-thirds (67.1%) of the participants from urban areas had a higher socioeconomic status (Class II/III) vis-à-vis 36.6% of participants from rural areas. None of the participants from rural areas were in the lower socioeconomic group (Class I) as against 18.1% of urban participants. On the contrary, almost one-fourth (25.2%) of the study subjects in rural areas had high socioeconomic status (Class V) in comparison to only 2% in urban areas (Figure 1).

**Figure 2:**
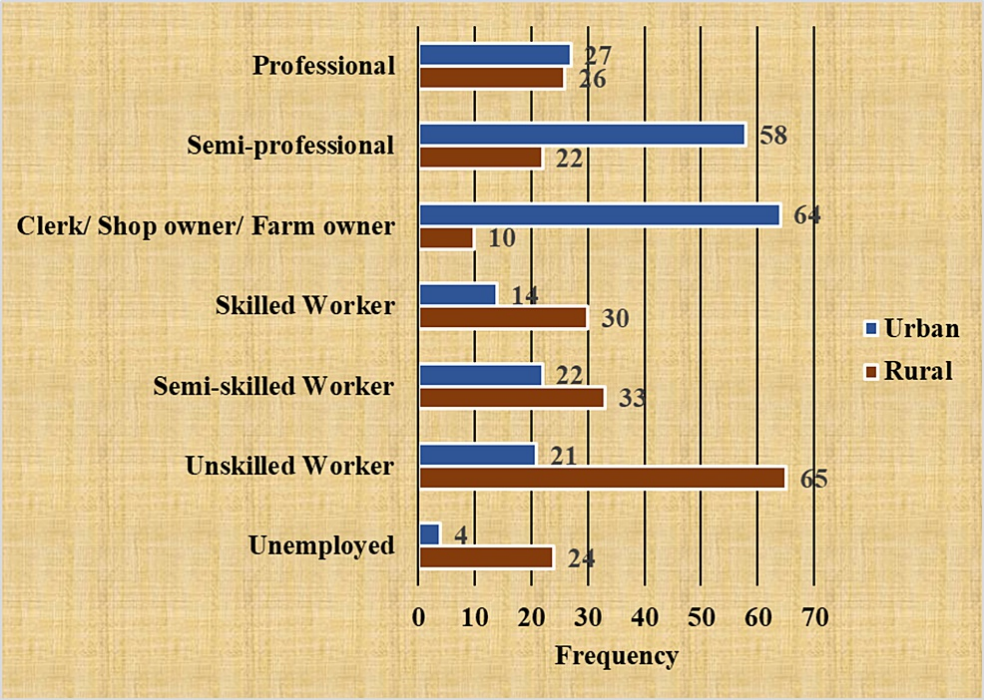
Distribution of study participants on the basis of the occupational status of the head of the family

In the present study, the heads of the families of almost one-third of participants in rural areas were unskilled workers, whereas, in urban areas, more than half (30.4%) of them were either clerks/shop owners/farm owners or semi-professionals (27.6%) (Figure 2).

Self-perception of health status

About two-fifths (41%) of participants in rural areas were unobservant of their health status, whereas 30% perceived that their health was poor. One in 10 participants perceived their health as very good; however, only a few (6%) perceived it as very poor. On the other hand, in the urban area, most of the adolescents perceived their health status as good (56%) and very good (33%). Only a few urban adolescents perceived their health as poor (5%) or very poor (1%). On examination, skin infections were the most common (37.2%) disease condition observed among males in rural areas followed by ear discharge (15.9%), whereas in urban areas, males more commonly had acne (39.4%) followed by refractive errors (26%). Among females, pallor (58.4%) followed by ear discharge and skin infections (46.1%) were seen in rural areas, whereas, pallor (31.8%) followed by refractive errors (21.9%) was seen in urban areas.

Prevalence of malnutrition

**Figure 3:**
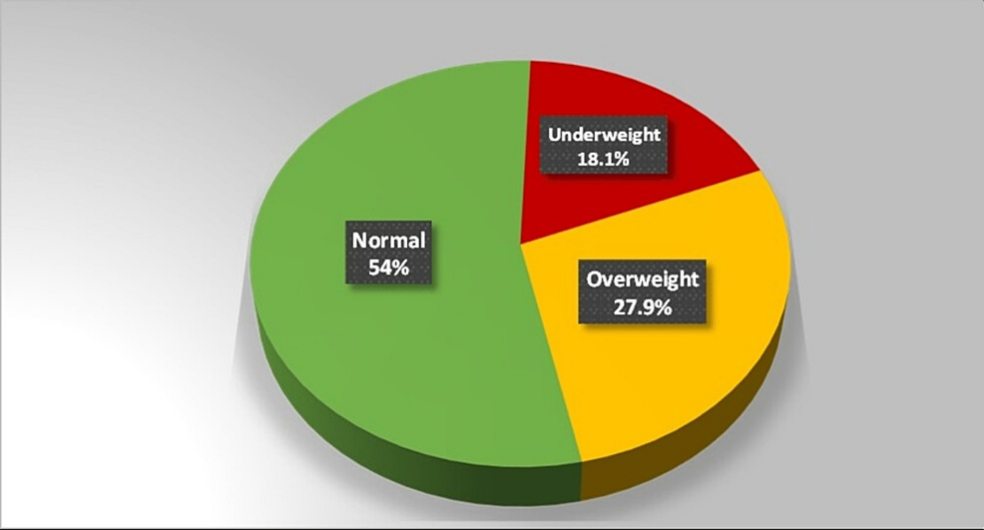
Distribution of study participants on the basis of nutritional status

The overall prevalence of malnutrition was calculated as 46%, wherein 18% of study subjects were undernourished and 28% were overnourished. Of the undernourished group, 13% were thin and 5% were severely thin; on the other end of the spectrum, 21 % were overweight and about 7% were obese (Figure 3).

Determinants of malnutrition

The prevalence of underweight was found to be approximately three times more in adolescents in rural (27.6%) as compared to urban areas (8.6%) while being overweight was more rampant in urban areas (42.4%) compared to rural areas (13.3%) (Table 2).

**Table 2:** Comparison of nutritional status of study participants from rural and urban areas

Residence	Nutritional Status			Total	χ 2, df, p-value
	Underweight	Normal	Overweight		
Rural “n” (%)	58 (27.6)	124(59.0)	28(13.4)	210(100.0)	54.799, 2, p = 0.002
Urban “n” (%)	18 (8.6)	103(49.0)	89(42.4)	210(100.0)	
Total “n” (%)	76 (18.1)	227 (54.0)	117(27.9)	420(100.0)	

The mean BMI of participants from urban areas (22.13 ± 2.65) was higher than the mean BMI of study subjects from rural areas (19.81±2.55). The difference was statistically significant as shown by an independent t-test (t (418) = -9.167, p=0.041). The mean BMI (kg/m^2^) for urban females (22.58±2.86) was higher than the mean BMI of rural females (19.13±2.58). The mean BMI for urban males (21.80±2.43) was also higher than the mean BMI of rural males (20.11±2.49) (Table 3).

**Table 3:** Comparison of the mean BMI of study participants from rural and urban areas based on gender

BMI	Gender	Rural	Urban
Mean ± SD	Female	19.13 (2.58)	22.58 (2.86)
	Male	20.11 (2.49)	21.80 (2.43)
	Total (N=420)	19.81(2.55)	22.13(2.65)

## Discussion

The overall prevalence of malnutrition was 46% in the present study with 18% of study subjects being underweight, whereas 28% were overweight. Of the underweight adolescents, almost 13% were thin and 5% severely thin, whereas 21% of participants were overweight and about 7% were found to be obese. The prevalence of undernutrition was approximately three times more in rural (27.6%) as compared to urban areas (8.6%), which may be attributed to gender bias, more physically strenuous lifestyle, and lack of knowledge about adequate calorie requirements among adolescents. Being overweight was more rampant in urban areas (42.4%) compared to rural areas (13.3%), which can be attributed to the easy availability of junk food, higher screen time, and the sedentary lifestyle of urban adolescents. The mean BMI for urban participants (22.13 ± 2.65) was higher than the mean BMI of rural study subjects (19.81±2.55), and the difference was statistically significant.

This was consistent with the findings in a cross-sectional study conducted by Gupta A et al. in the Shimla district of North India in 2012, which showed that about 41% of adolescents were suffering from malnutrition, out of which 33% were undernourished, 7.1% were overweight while 1.3% were obese [8].

Similar findings were observed in the study conducted by Pathak S et al. in 2018 among urban and rural school-going adolescents of Vadodara in which a statistically significant difference (p <0.001) was found between urban and rural groups for the distribution of BMI categories [9]. In their study, 63.6% of the urban children were found to be either obese or overweight as compared to only 8.9% of obese or overweight children in rural areas.

As per the current study, girls were more malnourished at both extremes, as they were more underweight, which is probably attributable to gender neglect or distorted eating habits to achieve a certain body image, and more overweight in urban settlements due to restrictions on physical activity as compared to boys. About one-fourth (25.1%) of the female participants were found to be underweight as compared to 14% of males, whereas at the other end, 36% of the female participants were either overweight or obese as compared to 23% of male study subjects. In the rural scenario, females were more underweight (44.6%) as compared to males (20%) while more males (13.8%) were overweight as compared to females (12.3%). The difference was statistically significant (p=0.001). While in urban settings, more females were in the underweight category (11%) as compared to males (6.7%). Urban females also had a higher frequency of being overweight (52.7%) as compared to males (34.5%). The difference was statistically significant (p=0.005).

In a cross-sectional descriptive study conducted by Adesina AF et al. in Nigeria among adolescents in 2010, it was observed that a higher number of females were significantly overweight and obese than males (p<0.05) [10]. The mean BMI for females (20.01±3.50 kg/m^2^) was higher than that of males (19.01±2.58 kg/m^2^) and the difference was statistically significant (p<0.001). This could be attributed to differential resource allocation and physical activity levels across both genders and was in coherence with the findings observed in our study.

Strengths

Since the research was carried out in both rural and urban adolescents, the findings of this study can be extrapolated to both rural and urban areas. 

Limitations

As part of the study took place during the COVID pandemic while the lockdown was in place with necessitated changes in lifestyle and routine, findings may vary slightly as compared to normal circumstances.

## Conclusions

Thus, the overall prevalence of malnutrition among adolescents was 46% in the present study, out of which 18% were found to be undernourished while 28% were over-nourished. The prevalence of undernutrition was approximately three times more in rural areas as compared to urban areas while being overweight was more rampant in urban areas compared to rural areas. Hence, there is a need for opportunistic screening of adolescents to detect malnutrition, as most of the adolescents found to be malnourished were ignorant about its risk factors and disastrous consequences on their health. Also, there is an urgent need to bolster ongoing National programs for adolescent health and nutrition.
